# Retrospective Study of the Safety and Efficacy of Anlotinib Combined With Dose-Dense Temozolomide in Patients With Recurrent Glioblastoma

**DOI:** 10.3389/fonc.2021.687564

**Published:** 2021-07-20

**Authors:** Lei She, Lin Su, Liangfang Shen, Chao Liu

**Affiliations:** ^1^ Department of Oncology, Xiangya Hospital, Central South University, Changsha, China; ^2^ Department of Clinical Pharmacology, Xiangya Hospital, Central South University, Changsha, China; ^3^ Institute of Clinical Pharmacology, Central South University, Hunan Key Laboratory of Pharmacogenetics, Changsha, China

**Keywords:** recurrent glioblastoma, anlotinib, dose-dense temozolomide, efficacy, survival, adverse events

## Abstract

**Purpose:**

The purpose of this study was to retrospectively analyze the safety and clinical efficacy of anlotinib combined with dose-dense temozolomide (TMZ) as the first-line therapy in the treatment of recurrent glioblastoma (rGBM).

**Patients and Methods:**

We collected the clinical data of 20 patients with rGBM. All patients received anlotinib (12 mg daily, orally for 2 weeks, discontinued for 1 week, repeated every 3 weeks) combined with dose-dense TMZ (100 mg/m^2^, 7 days on with 7 days off) until the disease progressed (PD) or adverse effects (AEs) above grade 4 appeared. Grade 3 AEs need to be restored to grade 2 before continuing treatment, and the daily dose of anlotinib is reduced to 10 mg. The patients were reexamined by head magnetic resonance imaging (MRI) every 1 to 3 months. The therapeutic effect was evaluated according to Response Assessment in Neuro-Oncology (RANO) criteria. The survival rate was analyzed by Kaplan-Meier survival curve analysis. The baseline of all survival index statistics was the start of anlotinib combined with dose-dense of TMZ. National Cancer Institute-Common Terminology Criteria Adverse Events version 4.0 (NCI-CTCAE 4.0) was used to evaluate AEs.

**Results:**

Twenty cases of rGBM were evaluated according to the RANO criteria after treatment with anlotinib and dose-dense TMZ, including five cases of stable disease (SD), thirteen cases of partial response (PR), one case of complete response (CR), and one case of PD. The median follow-up time was 13.4 (95% CI, 10.5–16.3) months. The 1-year overall survival (OS) rate was 47.7%. The 6-month progression-free survival (PFS) rate was 55%. In the IDH wild type group, the median PFS and median OS were 6.1 and 11.9 months, respectively. We observed that AEs associated with treatment were tolerable. One patient stopped taking the drug because of cerebral infarction. There were no treatment-related deaths.

**Conclusion:**

Anlotinib combined with dose-dense TMZ for the first-line therapy showed good efficacy in OS, PFS, ORR, and DCR in the treatment of rGBM, and the AEs were tolerant. Randomized controlled clinical trials investigating the treatment of rGBM with anlotinib are necessary.

## Introduction

Glioblastoma (GBM) is the most common and invasive primary nervous system tumor (1, 2). The current standard treatment is to maximize surgical resection based on preserving brain function and to use temozolomide (TMZ), concurrent chemoradiotherapy (CCRT), and adjuvant chemotherapy (AC) after surgery as soon as possible. Despite active treatment, the median overall survival (OS) time of GBM is only 14.6 months (95% CI = 13.2–16.8 months), the 2-year OS rate is 26.5% (95% CI = 21.2%–31.7%), the median progression-free survival (PFS) time is 6.9 months (95% CI = 5.8–8.2 months), and approximately 85% of patients relapse within 2 years (3). The prognosis of patients with recurrent GBM (rGBM) is still poor, with a median OS time of less than 6 months (4). Researchers have been exploring a safe and effective treatment for rGMB. However, there is no level I recommendation for the treatment of rGMB in current major guidelines and consensus.

The dependence of tumor growth on angiogenesis has been recognized, and there is an overexpression of vascular endothelial growth factor (VEGF) and its receptor in many malignant tumors, which provides an important theoretical basis for the development of antiangiogenic drugs and targeted therapies. Gliomas are rich in neovascularization and highly express VEGF, which directly affects the prognosis of patients.

In 2009, the FDA approved the anti-VEGF monoclonal antibody bevacizumab (BEV) in rGBM due to the results of the BRAIN study. Targeted drugs against VEGF receptor (VEGFR), including sunitinib, sorafenib, and apatinib, are also being actively investigated in clinical trials (5–8). Some therapeutic effects have been achieved, but there is still room for improvement. The REGOMA trial, randomized, multicenter, open phase II clinical trial involving 119 patients with rGBM, showed that the median OS of patients in the regorafenib group and the lomustine group were 7.4 and 5.6 months, respectively (*P* = 0.0009), which was encouraged in rGBM. Besides, molecular detection in the trial showed that the efficacy of regorafenib was not related to O6-methylguanine-DNA methyltransferase (MGMT) methylation and isocitrate dehydrogenase 1 (IDH1) status but may be related to regorafenib targets (VEGFR, PDGFR, FGFR, TIE2) (9).

Anlotinib hydrochloride is a small molecular multitarget tyrosine kinase inhibitor (TKI). It can effectively inhibit VEGFR, PDGFR, FGFR, c-Kit, and other kinases, that was the same as regorafenib. It has the dual effect of inhibiting tumor angiogenesis and growth (10–13). Therefore, anlotinib, as a new generation of multitarget TKIs, is better than first-generation TKIs in terms of efficacy and safety window and has broader clinical application prospects. A number of clinical trials have also confirmed that anlotinib has good efficacy and safety in non-small cell lung cancer, small cell lung cancer, soft tissue sarcoma, medullary thyroid carcinoma, and renal cell carcinoma (14–18). Anlotinib has good membrane permeability, good oral bioavailability, and the capability of breakthrough of brain blood barrier (19). In addition, its antivascular activity is stronger than that of sorafinib and sunitinib in the term of half inhibitory concentration (IC50). In a mouse model of human tumor xenotransplantation, anlotinib also showed activity against glioma (13). ALTER0303 analysis showed that anlotinib was effective in the treatment of advanced non-small cell lung cancer with brain metastasis and could effectively control the progression of intracranial metastasis. The subgroup analysis of the ALTER1202 study showed that anlotinib was also effective in the treatment of brain metastasis of small cell lung cancer (20, 21). Therefore, anlotinib has therapeutic potential in brain tumor. A meta-analysis was conducted to evaluate the efficacy and tolerance of TMZ in the treatment of rGBM. TMZ standard administration regimen was compared with three TMZ dose density administration regimens. The results showed that 7 days on/7 days off regimen could bring better survival benefits to patients (22). Many clinical studies have shown that adverse effects (AEs) to anlotinib are tolerant (12, 23). Some clinical trials of anlotinib in the treatment of rGBM are underway. At present, there are only two cases, and it has been reported that anlotinib has a good effect in the treatment of rGBM (24, 25). Therefore, we retrospectively analyzed 20 patients in our hospital to evaluate the efficacy and safety of anlotinib combined with dose-dense TMZ in rGBM.

## Materials and Methods

### Patient Characteristics

We retrospectively analyzed the data of 20 patients with rGBM at Xiangya Hospital of Central South University from October 2019 to November 2020. The inclusion criteria included the following: 1) Karnofsky performance scale (KPS) ≥ 60; 2) ≥18 years old; 3) pathologically confirmed as GBM World Health Organization Grade IV; 4) the evaluation of recurrence by surgeons, radiologists, and oncologists according to the Response Assessment in Neuro-Oncology (RANO) criteria after magnetic resonance imaging (MRI) reexamination, including MR spectroscopy(MRS) and perfusion imaging; 5) measurable lesions; 6) good bone marrow, liver, and renal function; 7) recurrence treated with anlotinib combined with dose-dense TMZ; and 8) previously standard CCRT and AC. The exclusion criteria were as follows: 1) complications with other malignant tumors or serious diseases; 2) patients with hypertension who cannot be reduced to the normal range after antihypertensive drug treatment. Patients with grade I or above myocardial ischemia or myocardial infarction, arrhythmia (including QT interval ≥ 440ms), and class II cardiac insufficiency; arteriovenous thrombosis events occurred within 6 months, such as cerebrovascular accidents (including temporary ischemic attacks), deep venous thrombosis, and pulmonary embolism; and 3) lack of follow-up data. The clinical data collected included sex, age, history of operation, pathological type, history of radiotherapy, history of chemotherapy, start and end time of anlotinib treatment, combined therapy, dosage, therapeutic toxicity, and reduction. The changes in bone marrow, liver and kidney function, urinary protein, blood pressure, skin of hands and feet, thyroid function, and seizure were recorded before and after each treatment cycle. Patients were evaluated every two cycles of medication.

### Treatment Methods

All patients received anlotinib (12 mg daily, orally for 2 weeks, discontinued for 1 week, repeated every 3 weeks) combined with dose-dense TMZ (100 mg/m^2^ daily on a 28-day cycle, orally for 7 days on with 7 days off) until the disease progressed (PD) or AEs above grade 4 appeared. Grade 3 AEs need to be restored to grade 2 before continuing treatment, and the daily dose of anlotinib is reduced to 10 mg.

### Efficacy and Adverse Events

Therapeutic effect was evaluated according to the RANO standard. Routinely, MRI was scanned after every two cycles of TMZ treatment. However, two patients experienced headache or seizure during the treatment process. They underwent an MRI scan in advance. However, both patients also received MRI evaluation 1 month later. Another patient could not be admitted to our hospital in time because of the COVID-19 epidemic. MRI was examined in the local hospital instead, which demonstrated the tumor was stable and the treatment plan remained. The patient had not admitted to our hospital until 3 months after the last MRI reexamined in our hospital. Although the reexamined MRI intervals were 1 to 3 months, the time point of evaluation of effect was two cycles of TMZ treatment (about 2 months) in all patients. Clinicians assessed disease status as complete remission (CR), partial remission (PR), stable disease (SD), and PD based on MRI images and clinical symptoms. We defined the overall response rate (ORR) as the ratio of the total number of patients to the total number of patients reaching CR and PR and the disease control rate (DCR) as the ratio of the total number of patients to the total number of patients reaching CR, PR, and SD. AEs were evaluated according to the National Cancer Institute-Common Terminology Criteria Adverse Events version 4.0 (NCI-CTCAE 4.0). We define overall survival (OS) and PFS since the start of anlotinib combined with TMZ. The main endpoint of this study were 6-month PFS and 1-year OS.

### Statistical Analysis

SPSS23.0 statistical software was used. The baseline data and AEs data of the patients were calculated by the direct counting method, and the survival rate was analyzed by the Kaplan-Meier survival curve. The baseline of all survival index statistics was the start of anlotinib combined with dose-dense of TMZ.

### Ethics

This study was approved by the Ethics Committee of Xiangya Hospital of Central South University. We obtained informed consent for all patients, and we guarantee that the patient data will be kept confidential.

## Results

### Patient Characteristics

**Table 1** summarizes the characteristics of 20 patients with rGBM, including sex, age, Karnofsky performance scale (KPS), surgery types, pathological molecular features, and the use of targeted drugs before recurrence. The median age of these patients was 52 years, and males accounted for 50%. In terms of pathological classification, two patients were IDH1 positive, and eight patients had MGMT methylation. 90% patients accepted gross total surgery. Three patients (15%) had used targeted therapy, mainly nimotuzumab in the previous CCRT. We observed that all 20 patients were treated with dose-dense TMZ in addition to anlotinib for the first-line therapy after relapse.

**Table 1:** Clinical characteristics.

Characteristics	No. (n=20)	%
Age (years)		
Median (range)	52 (18~71)	
Sex		
Male	10	50
Female	10	50
KPS		
90-80	13	65
70-60	7	35
Surgery types		
Gross total surgery	18	90
Subtotal surgery	2	10
IDH1 Mutation Status at first diagnosis		
+	2	10
–	18	90
MGMT promoter at first diagnosis		
Methylation	8	40
Unmethylation	12	60
History of targeted therapy before recurrence		
Yes	3	15
No	17	85

KPS, Karnofsky performance scale; IDH1, isocitrate dehydrogenase 1; MGMT, O6-methylguanine-DNA methyltransferase; TMZ, temozolomide.

### Efficacy

**Figure 1:**
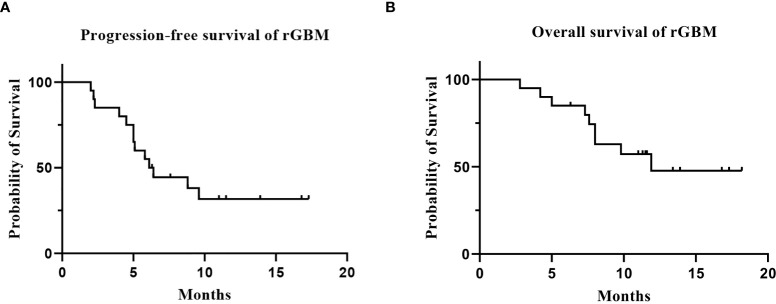
The survival analysis of the 20 patients with rGBM who received anlotinib and TMZ treatment. [**(A)** progression-free survival; **(B)** overall survival].

**Figure 2:**
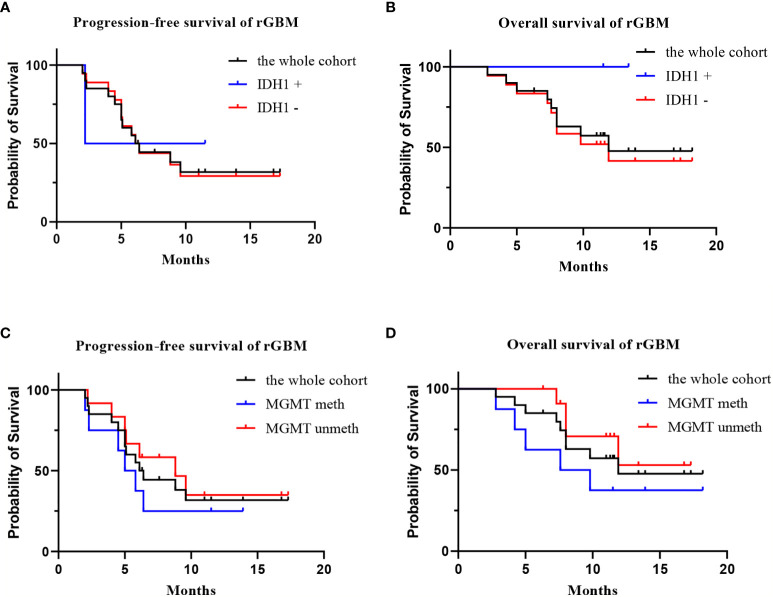
The survival analysis of the 20 patients with rGBM who received anlotinib and TMZ treatment. [**(A)** progression-free survival of IDH1+ group, IDH1−group, and the whole cohort; **(B)** overall survival of IDH1+ group, IDH1 –group, and the whole cohort; **(C)** progression-free survival of MGMT meth group, MGMT unmeth group, and the whole cohort; **(D)** overall survival of MGMT meth group, MGMT unmeth group, and the whole cohort].

**Figure 3:**
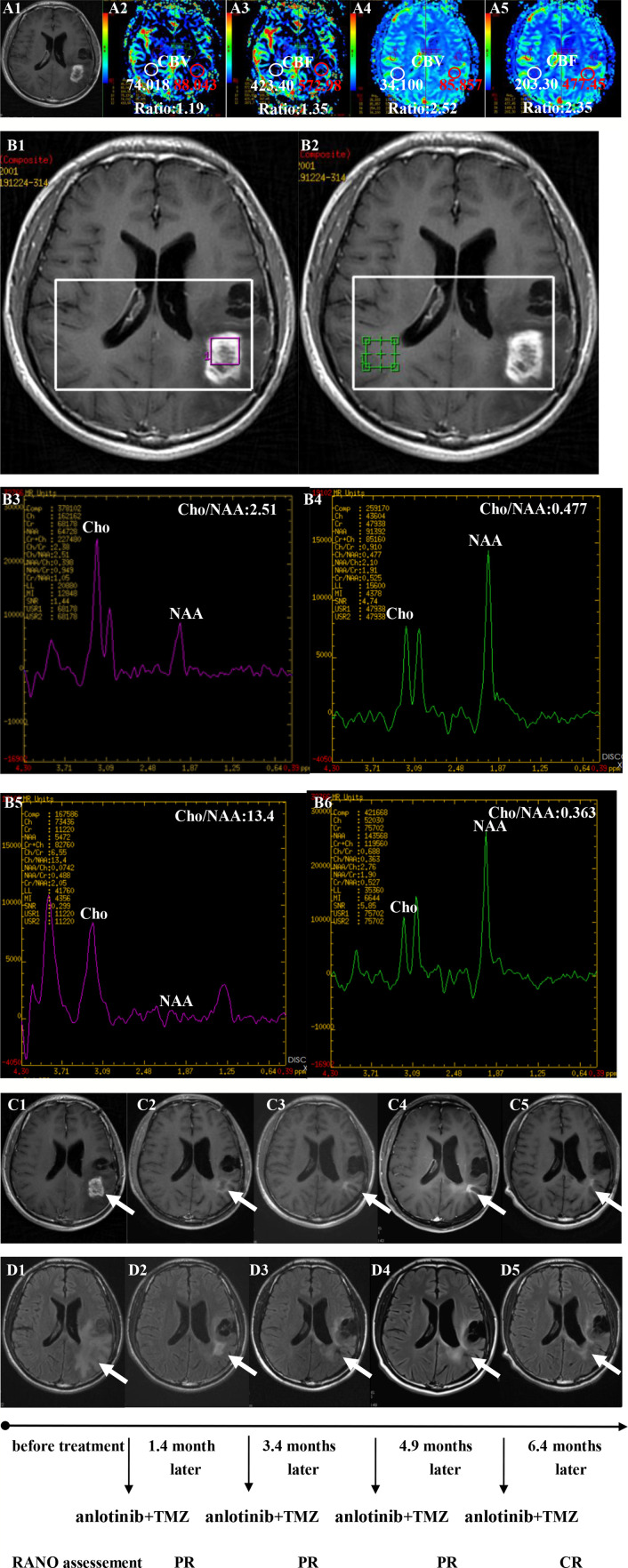
Brain scan of patient number 5 (IDH1−; in situ recurrence. The interval time between the end of CCRT and the treatment of anlotinib combined with TMZ was 3.8 months). **(A1–A3)** Perfusions maps at the time of suspected recurrence; **(A4, A5)** Perfusions maps at the time of diagnosis of recurrence 1.1months later, just watch and see, no additional therapy; **(B1–B4)** MR spectroscopy (MRS) at the time of suspected recurrence; **(B5, B6)** MR spectroscopy (MRS) at the time of diagnosis of recurrence 1.1 months later, just watch and see, no additional therapy; **(C1–C5)** Contrast-enhanced MRI; **(D1–D5)** MRI-Flair; MRI images followed-up every 1 to 3 months before and after treatment. The arrow refers to the area of recurrence. The patient achieved complete remission after treatment and had a progression-free survival time of 16.8 months.

**Figure 4:**
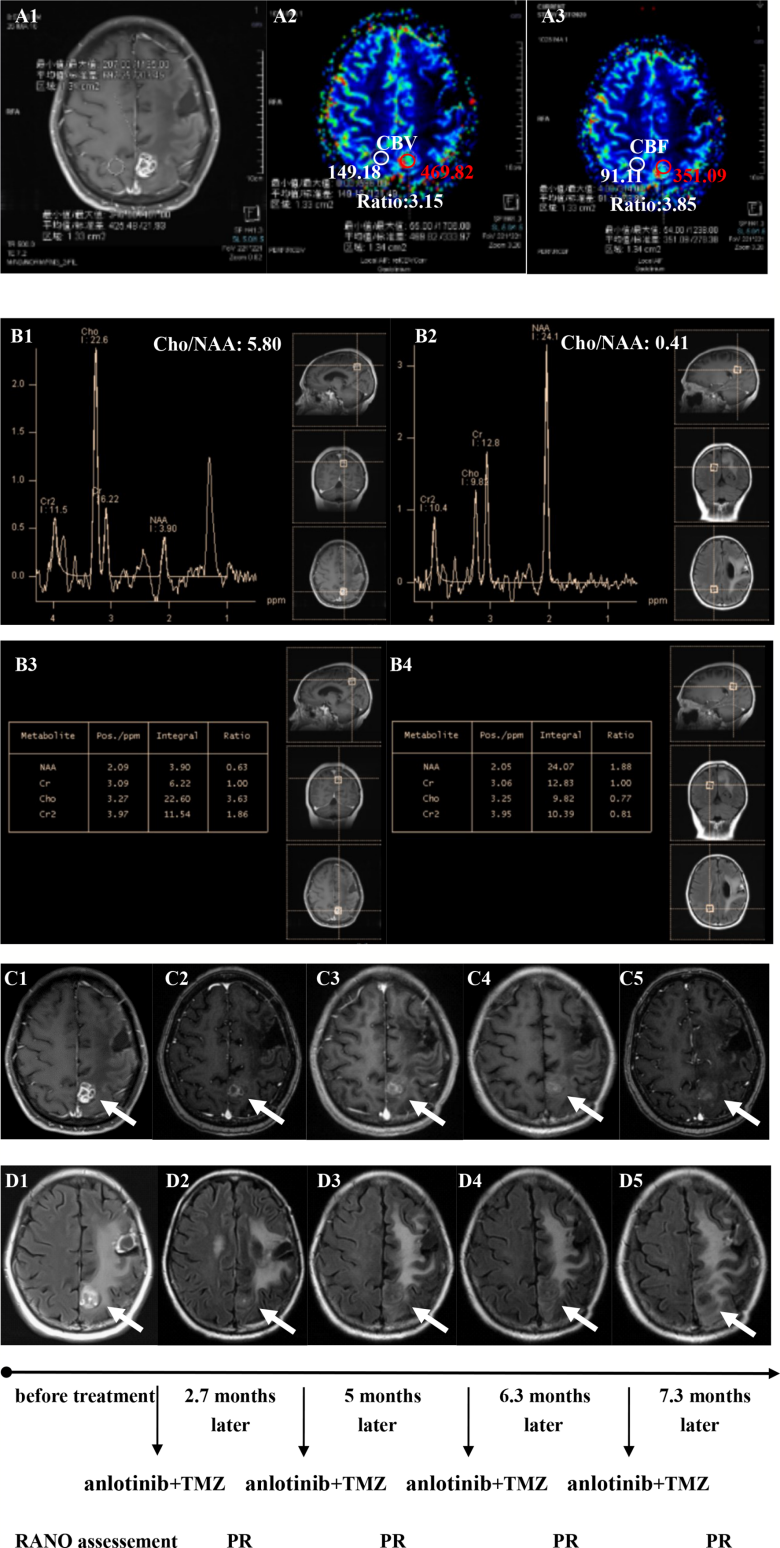
Brain scan of patient number 17 (IDH1−; Recurrence occurred at a second site; The interval time between the end of CCRT and the treatment of anlotinib combined with TMZ was 2.9 months). **(A1–A3)** Perfusions maps at the time of recurrence; **(B1–B4)** MRS at the time of recurrence; **(C1–C5)** Contrast-enhanced MRI; **(D1–D5)** MRI-Flair; MRI images followed-up every 1 to 3 months before and after treatment. The arrow refers to the area of recurrence. The patient achieved partial remission after treatment and had a progression-free survival time of 8.8 months.

**Figure 5:**
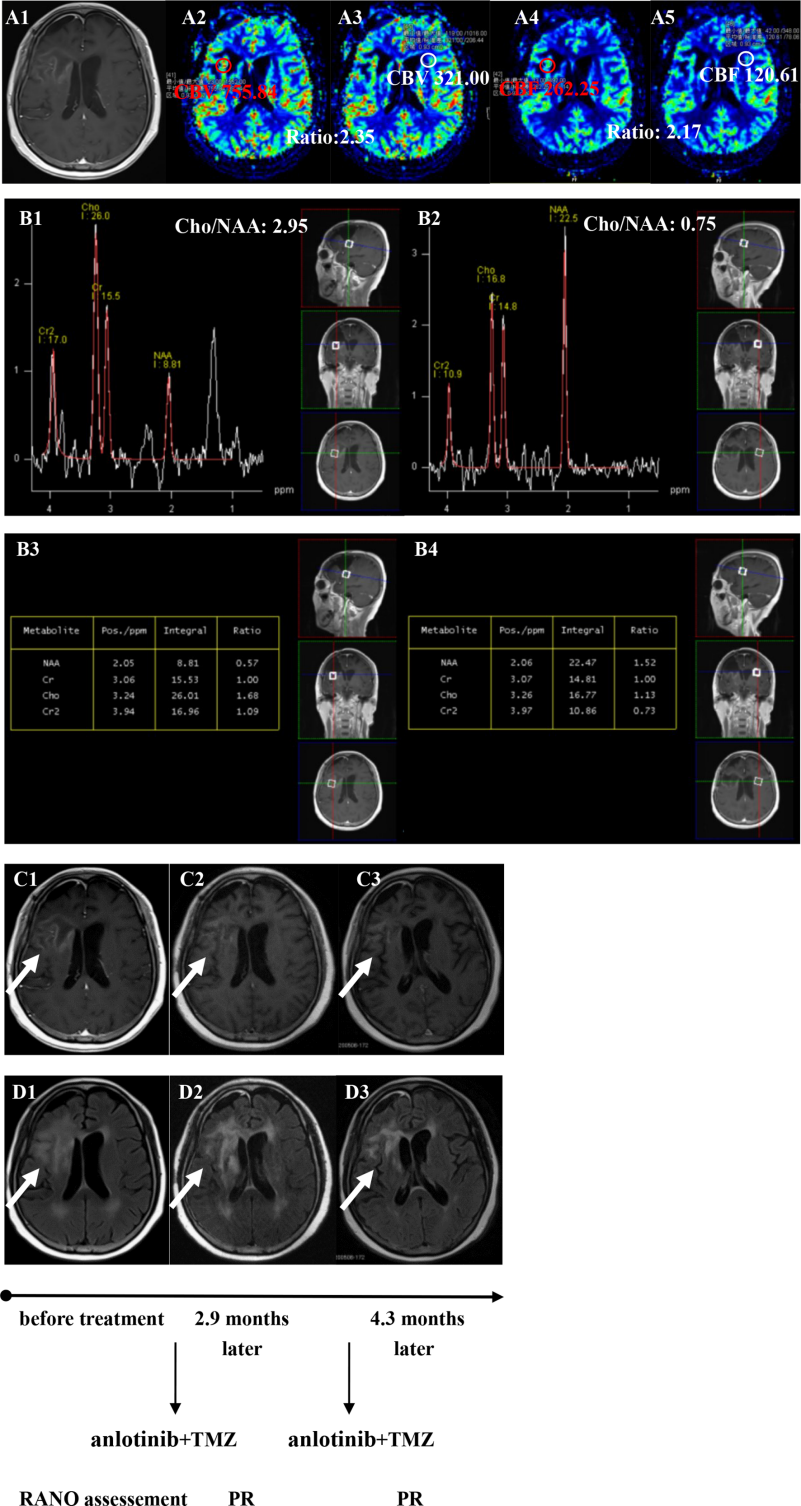
Brain scan of patient number 3 (IDH1−; in situ recurrence. The interval time between the end of CCRT and the treatment of anlotinib combined with TMZ was 3.2 months). **(A1–A5)** Perfusions maps at the time of recurrence; **(B1−B4)** MRS at the time of recurrence; **(C1–C3)** Contrast-enhanced MRI; **(D1–D3)** MRI-Flair; MRI images followed-up every 1 to 3 months before and after treatment. The arrow refers to the area of recurrence. The patient achieved partial remission after treatment and had a progression-free survival time of 5.8 months.

Twenty patients with rGBM were evaluated according to the RANO criteria after treatment with anlotinib plus TMZ, including five cases of SD, 13 cases of PR, one case of CR, and one case PD (**Table 2**); the ORR was 70% (14/20), and the DCR was 95% (19/20). Our median follow-up time was 13.4 (95% CI=10.5–16.3) months. The median interval time between the end of CCRT and the treatment of anlotinib combined with TMZ was 3.9 months. The median interval time between operation and recurrence was 6.7 months, which was consistent with EORTC 26981 study. All patients were treated with anlotinib as a first-line treatment after recurrence. We were highly vigilant of pseudoprogression before making a diagnosis of recurrence, especially for patients who were diagnosed as recurrence within 3 months after CCRT. According to the main points of differentiation between pseudoprogression and recurrence, such as resection extent of surgery, molecular pathology, time of recurrence, clinical symptoms, MRI-enhanced images, function MRI, including perfusion and spectrum, and so on. We organized surgeons, radiologists, and oncologists to conduct multi-disciplinary treatment. For patients with suspective-recurrence within about 3 months, we chose dynamic observation for a period and then re-examined MRI and function MRI, such as patient numbers 1, 3, 5, 16, and 17. Among them, one patient received subtotal resection because of motor area lesion, another patient recurrence occurred at a second site. The molecular pathology of all five patients were IDH-. Besides, the symptoms, such as limb numbness and weakness, worsened obviously in these patients. Even though we chose to watch and see, as time went by, these tumors were further enlarged after reexamination of MRI-enhanced images, and two patients received MRS and perfusion imaging, which showed that the perfusion ratio and Cho/Naa was higher than the first suspective-recurrence imaging. The symptoms of these five patients were improved after the use of anlotinib combined with TMZ. Some patients also received MRS and PWI after using the combined therapy, showed Cho/Naa and perfusion decreased sharply, further confirming the PD. In sum, the diagnosis of true progression was supported. After the diagnosis of PD and start of anlotinib and dose-dense TMZ, the reexamined MRI intervals were 1 to 3 months, the time point of evaluation of effect was two cycles of TMZ treatment (about 2 months) in all patients. **Table 3** records the detailed timeline of each patient’s treatment process. The 1-year OS rate was 47.7%. The 6-month PFS rate was 55%. The median PFS and median OS of these 20 patients were 6.1 months and 11.9 months respectively (95% CI = 4.9–7.3). The median PFS and median OS of patients with IDH1 wild type were 6.1 (95% CI=4.9–7.3) months and 11.9 (95% CI = 5.7–18.1) months, respectively. There was no significant difference in PFS and OS between IDH1+ and IDH1− groups. So were the patients in MGMT unmeth and MGMT meth groups (*P*=0.855; *P*=0.226; *P*=0.350; *P*=0.168, respectively). These data suggested the benefit of anlotinib combined with dose-dense TMZ therapy may be not associated with IDH and MGMT status. It is necessary to explore the specific benefit population based on molecular pathology. The survival curve is shown in **Figures 1** and **2**. **Figures 3**–**5** show the re-examination of MRI changes in patients with rGBM after treatment with anlotinib.

**Table 2:** Efficacy data (RANO criteria).

Best objective response	No. (n=20)	%
CR	1	5
PR	13	65
SD	5	25
PD	1	5

**Table 3:** Treatment timeline of 20 patients with rGBM.

Patient number	Time point of surgery	Time point of CCRT starting	Time point of CCRT ending	Time point of recurrence	Time point of treatment of anlotinib+TMZ	Time point of progress	Time point of death	Follow-up time
1	-4.3	-3.6	-2.1	-1.1	0	5	–	18.2
2	-8.3	-7.3	-5.8	0	0	6.1	11.9	11.9
3	-5.7	-4.7	-3.2	-1.7	0	5.8	7.6	7.6
4	-7.2	-6.2	-4.7	0	0	–	–	17.3
5	-6.8	-5.3	-3.8	-1.1	0	–	–	16.8
6	-5.2	-4.5	-3	0	0	5	8	8
7	-10.7	-9.9	-8.3	-1.3	0	2.3	4.2	4.2
8	-7.9	-6.1	-4.6	-0.1	0	4.5	5	5
9	-8.9	-7.6	-6.1	0	0	5.1	7.3	7.3
10	-6.5	-5.3	-3.8	-0.7	0	2.2	–	13.4
11	-6.4	-4.8	-3.3	0	0	6.4	9.8	9.8
12	-19.1	-16.8	-15.3	0	0	–	–	13.9
13	-23.1	-21.4	-19.8	-0.2	0	–	–	11.5
14	-11.6	-10.5	-9	-0.5	0	9.6	–	11.3
15	-11	-9.7	-8.2	0	0	–	–	11
16	-4.4	-3.1	-1.6	-0.1	0	4	8	8
17	-5.6	-4.4	-2.9	-2.1	0	8.8	–	11.6
18	-6.1	-5.3	-3.8	0	0	–	–	7.6
19	-6.3	-5.4	-3.9	-0.3	0	2	2.8	2.8
20	-9.2	-8.0	-6.4	0	0	–	–	6.3
median	-6.8	-5.4	-3.9	-0.1	0	6.1	11.9	13.4

Take ‘Time point of treatment of anlotinib+TMZ’ as the starting point of the timeline, denoted by 0, and the rest of the column is the interval time (months) of distance 0. -means no progress and no death.

**Timepoint of surgery:** The interval time point between the treatment of anlotinib combined with temozolomide (anlotinib+TMZ) and the first surgery.

**Timepoint of CCRT starting:** The interval time point between the treatment of anlotinib+TMZ and the start of concurrent chemoradiotherapy (CCRT).

**Time point of CCRT ending:** The interval time point between the treatment of anlotinib+TMZ and the end of CCRT.

**Timepoint of recurrence:** The interval time point between the treatment of anlotinib+TMZ and the disease recurrence.

**Timepoint of treatment of anlotinib+TMZ:** The time point at which the patients began to be treated with anlotinib+TMZ. We define it as 0.

**Timepoint of progress:** The interval time point between the disease progression and the treatment of anlotinib+TMZ.

**Timepoint of death:** The interval time point between the death and the use of anlotinib+TMZ.

**Follow-up time:** The time from the beginning of the treatment of anlotinib+TMZ to the present or death.

### Adverse Events

According to the ALTER0303 phase III clinical study and Anlotinib Adverse Reaction Management Manual, the AEs of anlotinib mainly include fatigue, gastrointestinal reactions, oral mucositis, hypertension, hand-foot skin reactions, rash, proteinuria, hypothyroidism, bone marrow suppression, abnormal liver and kidney function, and abnormal blood lipids. However, the only AEs we observed in these 20 patients were hypertension, rash, hand and foot skin reaction, mouth ulcers, thrombocytopenia, an increase in liver enzymes, and an increase in triglycerides (**Table 4**). Mild AEs could be tolerated after symptomatic treatment. Two patients were reduced from 12 to 10 mg because of grade 3 hypertension and grade 3 hand-foot skin reaction, and one patient stopped taking the drug because of cerebral infarction and seizure. There were no treatment-related deaths.

**Table 4:** Analysis of adverse events.

Adverse Events	Grade 1 or 2 (n,%)	Grade 3 or 4 (n,%)	Total (n,%)
Hypertension	1 (5)	4 (20)	5 (25)
Rash	1 (5)	0 (0)	1 (5)
Hand-foot skin reaction	3 (15)	1 (5)	4 (20)
Mouth ulcers	1 (5)	1 (5)	2 (10)
Thrombocytopenia	1 (5)	0 (0)	1 (5)
Increase in liver enzymes	1 (5)	0 (0)	1 (5)
Increase intriglycerides	1 (5)	0 (0)	1 (5)
Cerebral infarction	0	1 (5)	1 (5)

## Discussion

At present, the standard treatment for GBM is surgical resection followed by standardized radiotherapy and chemotherapy. Although approximately 85% of patients relapse within 2 years. The prognosis of these patients is poor after recurrence, and the median OS time is 6 to 12 months. There is no high-level evidence for the treatment of rGBM. Central recurrence is the main recurrence mode of high-grade glioma (26). Large-scale re−radiotherapy or short-term re−irradiation may cause serious side effects, such as radiation necrosis. The current evidence for repeated radiotherapy in recurrence is based on single-arm prospective or retrospective studies. Whether using stereotactic radiosurgery, fractionated stereotactic radiotherapy, or traditional fractionated radiotherapy, the median PFS and OS of rGBM are not optimistic (27–29). Besides, the value of chemotherapy in rGBM is uncertain. Regardless of TMZ intensive chemotherapy regimen is used after relapse or TMZ is given in any period of recurrence, including early group, extended group, rechallenge group, the 6-month PFS rate of rGBM remains less than 35% (22, 30).

In recent years, some clinical researches on targeted therapy have been carried out for rGBM. In addition to the use of BEV, targeting other signaling pathways, such as EGFRV III, mTOR, integrin, EGFR antibody, PARP receptor inhibitor (31–34), has achieved limited efficacy.

GBM is rich in neovascularization and highly expresses VEGF, which contribute to tumor growth and metastasis. Many preclinical studies have proven that antiangiogenic drugs can significantly inhibit tumor cell growth and metastasis. Antiangiogenic therapy has shown clinical benefits in a variety of tumors in many clinical trials. BEV, a monoclonal antibody against VEGF, was approved by the FDA in 2009 for the treatment of rGBM since the uncover of the BRAIN study. In 2017, the efficacy of BEV combined with lomustine (Lom) in 437 patients with rGBM compared with Lom alone was evaluated in the EORTC26101 study. The combined Bev regimen prolonged the median PFS time from 1.5 to 4.2 months (HR = 0.49, 95% CI = 0.39–0.61 months) but did not significantly improve the OS time (9.1 months *vs*. 8.6 months) (35). Clinical trials and data from Chen et al. also showed that combining Lom with BEV improved ORR and PFS but not OS in patients with rGBM (36). In recent years, tyrosine kinase inhibitors (TKIs), which inhibit angiogenesis, have also been evaluated in patients with rGBM. A single-arm II phase multicenter trial included 40 patients with rGBM who took sunitinib daily. The results showed that the median PFS was 2.2 months, and the median OS was 9.2 months (7). A noncontrolled open study explored the efficacy of apatinib combined with TMZ in the treatment of rGBM. The results showed that both PFS and OS improved, with a median PFS of 6 months (95% CI = 5.3–7.8 months) and a median OS of 9 months (95% CI = 8.2–12.2 months) (37). Cediranib is a TKI of VEGFR2, C-Kit, and PDGFR. A phase II clinical trial showed an encouraging 25.8% PFS rate with cediranib (38). The REGOMA trial, published in the Lancet on December 3, 2018, was a randomized, multicenter, open phase 2 clinical trial involving 119 patients. The results showed that the median OS times of patients in the regorafenib group and lomustine group were 7.4 months (95% CI = 5.8–12.0 months) and 5.6 months (95% CI=4.7-7.3 months), respectively (risk ratio = 0.50, 95% CI=0.33–0.75; *P*=0.0009). In addition, the study also demonstrated a significant improvement in the DCR and 6-month PFS in the regorafenib group compared with the lomustine group. Regorafenib has an encouraging OS advantage in rGBM and maybe a new potential therapeutic drug (9).

Anlotinib is a multi-target TKI, whose action target is similar to regorafenib, which can inhibit VEGFR, PDGFR, FGFR, c-Kit, and other pathways and finally inhibit angiogenesis. Anlotinib hydrochloride has a significant inhibitory effect on VEGFR2 and VEGFR3 at molecular level, and its IC50 is 0.2 and 0.7 nmol/L, respectively. The inhibitory effect on VEGFR2 is 20 and 575 times as much as that of sunitinib and sorafenib, and the inhibitory effect on VEGFR3 is 22 and 506 times that of sunitinib and sorafenib, respectively. Anlotinib has high activity for c-Kit, PDGFR α family and β family, and FGFR family 1–4 (13). After oral administration of anlotinib, the concentration could be detected in the cerebrospinal fluid of rats and in the cerebrospinal fluid of patients with brain metastasis of lung cancer. According to the results of the previous first-phase dose climbing test of anlotinib, combined with tolerance and efficacy, the recommended dose is 12 mg for 2 weeks and stops for 1 week. In rGBM, we refer to the approved doses of lung cancer and soft tissue sarcomas, and combined with two cases of anlotinib in the treatment of rGBM, the treatment method in our study is 12 mg, taking 2 weeks and stopping for 1 week (12, 39, 40).

The purpose of this study was to explore the efficacy and safety of anlotinib combined with dose-dense TMZ in the treatment of rGBM. To date, this is the first retrospective study in the world to analyze the efficacy and safety of anlotinib combined with dose-dense TMZ in the treatment of rGBM. In our study, one patient achieved CR, and thirteen patients achieved PR, with an ORR of 70% (14/20). There were five patients with SD, so the DCR was 95% (19/20). In addition, the 6-month PFS rate was 55%. The 1-year overall survival (OS) rate was 47.7%. In this study, the efficacy of anlotinib combined with TMZ in the treatment of rGBM was slightly higher than that of Wang et al. in their phase II clinical trial of apatinib combined with TMZ in the treatment of rGBM. In their study, the patients’ ORR was 45% (9/20), and DCR was 90% (18/20). The median PFS was 6 months, and the median OS was 9 months. However, retrospective study has inherent deficiencies, such as selective bias (37). The median follow-up time in our study was only 13.4 months, However, two patients who had no mutation in IDH1 in our study were followed up for more than 15 months, during which their condition remained stable, and there was no progression. This finding is also encouraging.

Regarding tolerability and compliance, in our study, the most common AE was hypertension (25%), which is consistent with apatinib in the treatment of rGBM and with anlotinib treatment of other tumors (33, 37). Other AEs included hand and foot skin reaction (20%), rash (5%), mouth ulcers (10%), thrombocytopenia (5%), an increase in liver enzymes (5%), an increase in triglycerides (5%), and cerebral infarction (5%). Almost all AEs are graded below 3 except one experienced cerebral infarction and the other two had grade 3 AEs. As this study is retrospective, some AEs may have occurred, which were not recorded during the study period. There were no treatment-related deaths. The dosage of anlotinib in our study was 12 mg, which refers to the dosage of anlotinib in non-small cell lung cancer and soft tissue sarcoma. The optimal dose of anlotinib in the treatment of rGBM is not clear, and a dose climbing test is needed.

The treatment of rGBM is still challenging. Although some clinical progress has been made in antiangiogenic therapy, the actual clinical effect is far from expected. At present, no effective targeted drug has been proven to prolong the OS of patients. This may be related to drug resistance, lack of predictive biomarkers, and failure to choose drugs under the guidance of accurate second-generation sequencing. With the deepening of clinical and basic research on antiangiogenic therapy of glioma, multitarget antiangiogenic drugs have certain development prospects. Our study is a retrospective, small sample study and can only provide a prospect for the treatment of rGBM. At present, several centers in China have conducted prospective randomized controlled clinical trials of anlotinib in the treatment of rGBM. It is believed that with the further development of clinical research, anlotinib can bring more hope for the clinical treatment of patients with malignant glioma. It is also expected that accurate biomarkers can be screened at the genetic level to identify patients sensitive to anlotinib.

## Conclusions

In summary, anlotinib combined with dose-dense TMZ for the first-line therapy is reliable, safe, and effective in the treatment of rGBM. In the future, we will conduct prospective clinical trials to further confirm the clinical value of anlotinib combined with dose-dense TMZ in rGBM.

## Data Availability Statement

The raw data supporting the conclusions of this article will be made available by the authors, without undue reservation.

## Ethics Statement

The studies involving human participants were reviewed and approved by Ethics Committee of Xiangya Hospital Central South University. The patients/participants provided their written informed consent to participate in this study. Written informed consent was obtained from the individual(s) for the publication of any potentially identifiable images or data included in this article.

## Author Contributions

LeiS wrote the first draft of this article. LinS and LiaS edited the first draft. CL approved the submitted version. All authors contributed to the article and approved the submitted version.

## Funding

This work was supported by the Nature Science Youth Foundation of Hunan Province (2018JJ3856).

## Conflict of Interest

The authors declare that the research was conducted in the absence of any commercial or financial relationships that could be construed as a potential conflict of interest.
